# Vocal fold immobility after thyroidectomy with intraoperative recurrent laryngeal nerve monitoring

**DOI:** 10.1590/S1516-31802007000300011

**Published:** 2007-05-03

**Authors:** Irene de Pedro Netto, Jose Guilherme Vartarian, Pablo Rodrigo Rocha Ferraz, Priscila Salgado, Juliana Bueno Meirelles de Azevedo, Ronaldo Nunes Toledo, José Ricardo Gurgel Testa, Elisabete Carrara-de-Angelis, Luiz Paulo Kowalski

**Keywords:** Thyroid gland, Thyroidectomy, Recurrent laryngeal nerve, Vocal cord paralysis, Intraoperative monitoring, Glândula tireóide, Tireoidectomia, Nervo laríngeo recorrente, Paralisia das cordas vocais, Monitorização intra-operatória

## Abstract

**CONTEXT AND OBJECTIVE:**

Intraoperative nerve monitoring has emerged as a valuable tool to facilitate recurrent laryngeal nerve identification during thyroid surgery, thereby avoiding its injury. The aim was to evaluate vocal fold mobility in patients who underwent thyroidectomy with intraoperative nerve monitoring.

**DESIGN AND SETTING:**

Cohort formed by a consecutive series of patients, at a tertiary cancer hospital.

**METHODS:**

The subjects were patients who underwent thyroidectomy using intraoperative laryngeal nerve monitoring, between November 2003 and January 2006. Descriptive analysis of the results and comparison with a similar group of patients who did not undergo nerve monitoring were performed.

**RESULTS:**

A total of 104 patients were studied. Total thyroidectomy was performed on 65 patients. Vocal fold immobility (total or partial) was detected in 12 patients (6.8% of the nerves at risk) at the first postoperative evaluation. Only six (3.4% of the nerves at risk) continued to present vocal fold immobility three months after surgery. Our previous series with 100 similar patients without intraoperative nerve monitoring revealed that 12 patients (7.5%) presented vocal fold immobility at the early examination, and just 5 (3.1%) maintained this immobility three months after surgery, without significant difference between the two series.

**CONCLUSION:**

In this series, the use of intraoperative nerve monitoring did not decrease the rate of vocal fold immobility.

## RESUMO

**CONTEXTO E OBJETIVO:**

A monitorização intra-operatória de nervos surgiu como uma ferramenta valiosa para facilitar a identificação do nervo laríngeo recorrente durante a cirurgia de tireóide, evitando a sua lesão. O objetivo foi avaliar a mobilidade das pregas vocais em pacientes submetidos a tireoidectomia com monitorização intra-operatória do nervo laríngeo recorrente.

**TIPO E LOCAL DO ESTUDO:**

Coorte de uma série consecutiva de pacientes em um hospital terciário de tratamento de câncer.

**MÉTODOS:**

Pacientes foram submetidos à cirurgia de tireóide usando a monitorização intra-operatória do nervo laríngeo recorrente, entre novembro de 2003 e janeiro de 2006. Uma análise descritiva dos resultados e uma comparação com um grupo similar de pacientes que não foram submetidos a monitorização dos nervos foram realizadas.

**RESULTADOS:**

Um total de 104 pacientes foi estudado. Tireoidectomia total realizada em 65 pacientes. Imobilidade de pregas vocais (parcial ou total) foi detectada em 12 pacientes (6.8% dos nervos sob risco) na primeira avaliação pós-operatória. Apenas 6 (3.4% dos nervos sob risco) permaneceram com imobilidade de prega vocal três meses após a cirurgia. Nossa série prévia com 100 pacientes similares sem a monitorização intra-operatória revelou que 12 pacientes (7.5%) apresentaram imobilidade de prega vocal na avaliação precoce, e apenas 5 (3.1%) mantiveram a imobilidade três meses após a cirurgia, sem diferença significativa entre as séries.

**CONCLUSÃO:**

Nesta série, o uso da monitorização intra-operatória do nervo laríngeo recorrente não diminuiu a taxa de imobilidade de prega vocal.

## INTRODUCTION

Thyroidectomy is one of the most frequent head and neck surgical procedures worldwide.^1^ Among the potential surgical complications, recurrent laryngeal nerve (RLN) injury with consequent vocal fold immobility may be a debilitating complication leading to voice changes. It may also give rise to the risk of respiratory distress and potential risk of laryngeal aspiration, thus leading to significant negative impact on patients’ daily lives.^1,2–5^

Injury to the laryngeal nerve may be secondary to direct trauma, unintentional sectioning, stretching, ligature entrapment or thermal or electrical injury.^2^ The increasing of the surgical team's experience, together with careful dissection and direct viewing of the RLN, has been considered to be the best approach towards avoiding such injuries.^3,6^

In recent years, the development of intraoperative laryngeal nerve monitoring has emerged as a valuable tool for facilitating RLN identification, with the aim of decreasing nerve injuries.^7^ However, there is a lack of prospective randomized trials for confirming such assertions. Several reports have described the benefits of intraoperative laryngeal nerve monitoring in patients who underwent thyroid surgery, particularly in cases of reoperation and large goiters.^3,4,8^ Nevertheless, there are other series that have failed to confirm such benefits.^2,5,9^

## OBJECTIVE

The objective of this study was to evaluate vocal fold mobility outcomes in patients who underwent thyroidectomy using intraoperative laryngeal nerve monitoring and compare the results with a previous series of similar patients without nerve monitoring, at the same institution.

## METHODS

This was a prospective study on a consecutive series of previously untreated adult patients who underwent thyroid surgery using intraoperative laryngeal nerve monitoring at our institution between November 2003 and January 2006. Videolaryngoscopic examinations were performed on all patients before surgery and at the times of 15 days, one month and three months after surgery.

Patients who had undergone previous head and neck surgical treatment, patients with voice complaints and patients found to have laryngeal abnormalities in the preoperative videolaryngoscopic evaluation were excluded from this study.

The laryngoscopic examination (with *Atmos Endo-Stroboscope 6*; color video monitor Sony-Trinitron PVN 1390, Lenzkirch, Germany; Sony videocassette recorder SVO-1410 HQ, Tokyo, Japan) was performed to detect either the presence or absence of vocal fold lesion and mobility, and the laryngeal configuration.

Demographic, clinical and surgical variables were extracted from the patient's medical files. In most cases, the surgical procedure was performed by 3^rd^ and 5^th^ year surgical oncology residents under direct supervision and assistance from the institution's attending head and neck surgeon. The surgical procedure followed the routine technique employed by the institution's Head and Neck Surgery Department, which included identification of parathyroids and RLN before ligation of the inferior thyroid pedicle and individual dissection and ligation of the vessels of the superior pole in order to avoid injury to the external branch of the superior laryngeal nerve. On a routine basis, there is no use of adjunctive tools such as magnifying glasses during the procedure.

To identify the RLN, the intraoperative nerve monitoring equipment used was the Medtronic Xomed Nerve Integrity Monitor-2^®^ (NIM-2; Jacksonville, Florida) electromyo-graphic endotracheal tube. This endotracheal tube contains electrodes along its wall that are placed against the true vocal folds. The grounding wires were placed in the subcutaneous tissue of the presternum area, and the nerve was stimulated with the Medtronic Xomed Prass monopolar nerve stimulator, usually adjusted to 1.0 mA. When the nerve was directly touched by the nerve stimulator, a characteristic audible beep confirmed its identification, and the electromyographic signal was recorded in the monitoring device (Medtronic NIM-Response). The use of this device helped in identifying the recurrent laryngeal nerve, sometimes even before directly viewing it. This decreased the need for extensive dissections and prolonged manipulations over and around the nerve, which theoretically would lead to reduced rates of nerve injury. Also, its use allowed identification of any anatomical variation in the nerve, such as nerve bifurcation and non-recurrent laryngeal nerve, and made it possible to record that the nerve was intact at the end of the procedure. The criterion for using nerve monitoring was convenience: it was used in patients whose health insurance plans covered the costs of the device. Because of this, there were no sample losses from this study.

A total of 104 patients were eligible for this study. There were 96 female patients (92.3%), and the patients’ ages ranged from 19 to 84 years (median: 43). Twelve patients (11.5%) reported tobacco use.

Total thyroidectomy was performed on 65 patients (62.5%), resulting in a total of 169 nerves at risk. Four patients (4.9%) underwent paratracheal lymph node dissection (three unilateral and one bilateral) and just one patient (1.0%) underwent a modified radical neck dissection (levels II to V). The duration of the procedure ranged from 100 to 310 minutes (median: 162.5 minutes).

The nodule sizes ranged from 0.3 to 5.0 cm (median: 1.5 cm). The final histology diagnosis revealed 47 cases (45.2%) of well-differentiated carcinomas, 40 (38.5%) of nodular goiters and nine (8.7%) of thyroiditis, and there were other benign conditions in eight patients (7.7%).

Postoperative surgical complications occurred in nine patients (8.7%). Temporary hypoparathyroidism was the most frequent complication and occurred in five patients (4.8%).

The possibility of associations between vocal fold immobility and certain demographic, clinical and surgical variables was investigated. The variables assessed were the following: gender, age (> 60 years), tobacco use, nodule size (> 3.0 cm), malignant histology, partial or total thyroidectomy, paratracheal neck dissection, procedure duration (> 160 minutes) and level of intubation difficulty based on the anesthesiologist's report.

This study was conducted after obtaining approval from the institution's Ethics Committee and all patients signed a consent form.

Descriptive analysis was performed. To investigate associations between independent variables, the chi-squared test and Fisher's exact test were used. The results were also compared with the findings from a previous consecutive series^10^ of similar patients who underwent thyroid surgery without laryngeal nerve monitoring, performed by the same surgical team at the same institution.

## RESULTS

In this study, alteration of vocal fold mobility (partial or total) was detected in 12 patients (11.2%), corresponding to 6.8% of the nerves at risk at the first postoperative evaluation. Out of these, only six patients (5.5%) continued to present altered vocal fold mobility at the evaluation three months after the surgery, corresponding to 3.4% of the nerves at risk.

Investigation of possible associations between vocal fold immobility and certain demographic, clinical and surgical variables showed that none of these variables were significantly associated with postoperative vocal fold mobility alteration (Table 1).

**Table 1.** Associations between demographic, clinical and surgical variables and the occurrence of vocal fold immobility in patients undergoing thyroid surgery with the use of intraoperative laryngeal nerve monitoring

Variable	Presence of vocal fold immobility No. of patients (%)		p-value*
	No	Yes	
Gender			
Male	7 (7.6)	1 (8.3)	0.929
Female	85 (92.4)	11 (91.7)	
Age			
≤ 60 years	87 (94.6)	11 (91.7)	0.530
> 60 years	5 (5.4)	1 (8.3)	
Tobacco use			
No	81 (88.0)	11 (91.7)	0.712
Yes	11 (12.0)	1 (8.3)	
Nodule size			
≤ 3.0 cm	85 (92.4)	10 (83.3)	0.277
> 3.0 cm	7 (7.6)	2 (16.7)	
Histology			
Benign	39 (42.4)	8 (66.7)	0.133
Malignant	53 (57.6)	4 (33.3)	
Intubation			
Easy	82 (89.1)	10 (83.3)	0.627
Difficult	10 (10.9)	2 (16.7)	
Extent of surgery			
Partial	37 (40.2)	2 (16.7)	0.203
Total	55 (59.8)	10 (83.3)	
Paratracheal dissection			
No	88 (95.7)	11 (91.7)	0.465
Yes	4 (4.3)	1 (8.3)	
Anesthesia duration			
≤ 160 minutes	46 (50.0)	6 (50.0)	1.000
> 160 minutes	46 (50.0)	6 (50.0)	

* *p = value obtained by chi-squared test or Fisher’s exact test.*

In five patients (4.8%), the RLN was visually identified during the surgery but this finding was not supported by an electromyographic response with the nerve stimulator. In these cases, the whole nerve monitoring system was checked and it was found that rotation of the endotracheal tube had occurred. The tube was then repositioned correctly for the proper setup and was found to function in all cases.

Our previous series^10^ of 100 similar patients (Table 2) who underwent thyroid surgery (58 total thyroidectomies, resulting in 158 nerves at risk) without intraoperative laryngeal nerve monitoring found that 12 patients (7.5% of the nerves at risk) presented vocal fold mobility alterations at the early examination (two weeks after surgery). Out of these, only five patients (3.1% of the nerves at risk) continued to present this immobility three months after surgery. Thus, there was no significant difference between the two series (p = 0.55).

**Table 2.** Demographic, clinical and surgical variables in the two series of patients

Variable	Category	Series	
		Previous series of patients^10^ (without nerve monitoring) n (%)	Present series of patients (with nerve monitoring) n (%)
Gender	male	12 (12.0)	8 (7.7)
	female	88 (88.0)	96 (92.3)
Age	median (years)	46	43
Tobacco use	no	92 (92.0)	92 (88.5)
	yes	8 (8.0)	12 (11.5)
Nodule size	< 3.0 cm > 3.0 cm	86 (86.0) 14 (14.0)	95 (91.3) 9 (8.7)
Histology	benign	54 (54.0)	57 (54.8)
	malignant	46 (46.0)	47 (45.2)
Intubation	easy	95 (95.0)	92 (88.5)
	difficult	5 (5.0)	12 (11.5)
Extent of thyroidectomy	partial total	42 (42%) 58 (58.0)	39 (37.5) 65 (62.5)
	no	91 (91.0)	99 (95.2)
Paratracheal neck dissection	yes	9 (9.0)	5 (4.8)
Anesthesia duration	median (minutes)	150	162.5

## DISCUSSION

Thyroidectomy is a very frequent surgical procedure for benign as well as for malignant thyroid disease, and more than 80,000 such operations are performed in the United States every year.^1^ RLN injury may be considered to be a debilitating complication that leads not only to voice disorders but also to respiratory distress and aspiration in some cases.^1,2–5^ Post-thyroidectomy voice alterations may have a significant impact particularly on professional voice users, and is a potential cause for litigation with medical-legal implications.^2,3,11^

Previous studies have reported rates of RLN injury that range from 0% to 4.8%,^12^ with higher rates in cases of large goiters, malignant histology and reoperations that may reach rates ranging from 2% to 12%.^5^ A retrospective historical series of 1020 patients who underwent thyroid surgery in our institution between 1990 and 2000 showed a vocal fold palsy rate of 1.8%.^12^ This rate is in accordance with other retrospective series.^1,13^ However, in prospective series where vocal fold mobility is actively assessed, higher rates have been reported in early evaluations.^9,14,15^ This was also found in our previous series that did not use nerve monitoring, in which there was an early RLN immobility rate of 7.5% that decreased to just 3.1% at the three-month follow-up.^10^

Careful dissection and viewing of the RLN and use of a more experienced surgical team are considered to be the best factors for avoiding RLN injury during thyroid surgery.^3,5,6,16^ A study by Sosa et al.^17^ reported on the cases of more than 5,800 patients who underwent thyroidectomy in which the surgeons were stratified according to the number of thyroid surgeries they performed. The study showed that the most experienced surgeons (with more than 100 thyroidectomies during the study period) had the lowest complication rates and achieved the shortest lengths of hospital stay, including the lowest RLN injury rate (0.4%). A prospective series published by Zambudio et al.^14^ confirmed such findings. In their series of 301 patients with multinodular goiter who underwent total thyroidectomy performed by experienced surgeons (prior accomplishment of at least 100 thyroidectomies), 26 cases (8.6%) presented RLN injury at the early evaluation, but only one patient (0.3%) continued to present such alteration at the long-term assessment.

Recently, the development of intraoperative laryngeal nerve monitoring has emerged as a valuable tool in facilitating RLN identification, which may lead to consequently decreased rates of nerve injury.^7^ There are several methods for monitoring the RLN, and all of these techniques involve stimulation of the RLN using a nerve stimulator and monitoring the response by means of electromyography of the voice muscle. The signal is converted into an acoustic signal that demonstrates the identification of the nerve.^8^ In addition, documentation of nerve integrity at the end of the surgical procedure could be used to lessen the legal repercussion of vocal fold immobility following surgery, by suggesting etiologies other than transection of the nerve.^2^ However, the value of this technique in relation to medical-legal issues should be addressed further.

Some reports have described the potential benefits of intraoperative laryngeal nerve monitoring in patients who underwent thyroid surgery.^3,4,8^ Such advantages are heightened in patients with large goiters or in situations of reoperation, which may give rise to extensive scarring of the surgical field and distortion of the dissection field.^4^ The largest series reported was the study by Thomusch et al.,^8^ which was a multicenter study of 4,382 patients who underwent thyroidectomy for benign goiter. In this study, two groups were compared: one group with visual RLN identification and the other with visual identification using intraoperative neuromonitoring. The rates of continuing RLN palsy were 0.8% and 0.4%, respectively, which was a significantly lower rate (p < 0.05) of RLN palsy for the neuromonitoring group. The authors concluded by recommending the use of such devices, and also stressed the advantage of documenting RLN identification.

On the other hand, there are other series that failed to confirm such benefits.^2,5,9^ Beldi et al.,^9^ studying 288 patients who underwent thyroidectomy using intraoperative nerve monitoring, found transient and continuing RLN palsy rates of 8.7% and 1.4%, respectively. They concluded that the incidence of RLN lesions during thyroid surgery was not decreased by the use of intraoperative neuro-monitoring. Witt^2^ reported on a retrospective survey of 136 patients who underwent thyroidectomy (a total of 190 nerves at risk) in which the rates of RLN palsy were compared between 107 unmonitored nerves and 83 monitored nerves. The unmonitored and monitored RLN presented continuing vocal fold palsy rates of 0.9% versus 2.4% (p > 0.05). Another interesting study was reported by Yarbrough et al.,^5^ in which they compared 52 cervical reexploration procedures that used intraoperative nerve monitoring, with 59 unmonitored cervical reoperations. They found no difference in unintentional continuing RLN palsy, with rates of 1.9% and 1.7% (p > 0.1), respectively.

It is important to note that, in several studies, electrophysiological RLN integrity did not always translate into clinical vocal fold mobility after surgery. This highlights the limited functional predictive value of intraoperative nerve monitoring.^2,5,9,15^

## CONCLUSIONS

In this series, the use of intraoperative laryngeal nerve monitoring in previously untreated patients who underwent thyroid surgery did not decrease the rate of vocal fold im-mobility, in comparison with a similar group of patients without the use of this monitoring. However, only a prospective randomized trial can better define its usefulness.
